# Canine idiopathic generalized tremor syndrome, immune-mediated?

**DOI:** 10.3389/fvets.2024.1453698

**Published:** 2024-09-20

**Authors:** Filip Kajin, Nina Meyerhoff, Sebastian Meller, Regina Carlson, Andrea Tipold, Rodrigo Gutierrez-Quintana, Adriana Kaczmarska, Daniel Sanchez-Masian, Edward Ives, Josep Brocal, Thilo von Klopmann, Julia Hauer, Holger Andreas Volk

**Affiliations:** ^1^Department of Small Animal Medicine and Surgery, University of Veterinary Medicine, Hannover, Germany; ^2^Clinic for Internal Diseases, Faculty of Veterinary Medicine, University of Zagreb, Zagreb, Croatia; ^3^Small Animal Hospital, School of Biodiversity, One Health and Veterinary Medicine, University of Glasgow, Glasgow, United Kingdom; ^4^Anderson Moores Veterinary Specialists, Hampshire, United Kingdom; ^5^Tierklinik Hofheim, Hofheim, Germany

**Keywords:** ataxia, autoimmune encephalitis, cerebellum, dog, immune-mediated, neural autoantibodies

## Abstract

Idiopathic generalized tremor syndrome is a disorder characterized by an acute onset of full-body tremors, sometimes accompanied by vestibulo-cerebellar signs, that is responsive to treatment with corticosteroids. Although considered to have an overall good outcome, relapsing and persistent mild clinical signs have been described. So far, little is known about the etiopathology of this syndrome, but it is believed to have an immune-mediated origin. In human medicine, description of numerous autoantibodies involved in certain non-infectious neurologic disorders has revolutionized understanding of their pathophysiology, diagnosis and treatment. In this multicenter retrospective study, we aimed to describe the clinical signs, course, and outcome of dogs with idiopathic generalized tremor syndrome and correlate potential findings with the presence or absence of autoantibodies associated with autoimmune cerebellar syndromes in humans. Information regarding signalment, history, clinical signs, laboratory findings, diagnostic imaging and testing for regional infectious diseases was gathered and the remaining serum and CSF samples were then analyzed for neural antibodies against targets associated with autoimmune encephalitic diseases of humans. Thirty-three dogs were included, and screening for neural antibodies was performed in 30 of those dogs. The analysis showed an increased titer of mGluR1 antibodies in two dogs, GFAP and later in the course of disease mGluR1 antibodies in one dog and an increase in unspecific autoantibodies which could not be further classified in two dogs. Dogs with detectable neural autoantibodies always had cerebrospinal fluid abnormalities in the form of a pleocytosis, with or without increased protein concentration, and tended to present with hyperthermia, potentially indicating a more severe clinical form of idiopathic generalized tremor syndrome in these cases. In conclusion, idiopathic generalized tremor syndrome is proposed to be an immune-mediated disorder potentially mediated by neural autoantibodies in a subgroup of dogs.

## Introduction

Immune-mediated meningoencephalitides are disease entities commonly reported in dogs, affecting mainly smaller, young-adult dog breeds (1, 2). Several different syndromes are recognized, including a generalized tremor syndrome and other encephalitides defined as granulomatous, necrotizing, mixed granulomatous and necrotizing or eosinophilic inflammatory patterns (1, 3–8). Idiopathic generalized tremor syndrome (IGTS) is characterized by an acute onset of full-body tremors that are responsive to treatment with corticosteroids (6). This disease is sporadically reported in the literature and known under many differing names, such as “Idiopathic cerebellitis,” “Corticosteroid-responsive tremor syndrome,” “Little white shaker syndrome” and “Steroid-responsive tremors” (6, 9, 10). Idiopathic generalized tremor syndrome was initially described in small (<15 kg), white dogs (e.g., Maltese, West Highland White terrier, Bichon Frise), and was at first introduced as “little white shaker syndrome” (9–11). However, various other dog breeds with different coat colors have since been reported, so the term IGTS may be more appropriate (7). Most dogs affected with this tremor syndrome are young (less than 5 years-old) and most weigh less than 15 kg (12). No gender predisposition has been documented, but a female bias has been suggested in some studies (6, 7). The disease occurs predominantly in dogs, with only a few cases described in cats (13). The prevailing clinical sign in dogs with this disease is a fine, whole-body tremor that worsens with anxiety and excitement, accompanied by cerebellar ataxia with hypermetria and wide-based stance and sometimes by vestibular signs (head tilt, nystagmus, vestibular ataxia), decreased menace response, opsoclonus, paraparesis, tetraparesis, mild hyperthermia and seizure activity (7, 12).

Pathologically, IGTS involves mild diffuse inflammation (i.e., lymphocytic meningoencephalitis), most pronounced in the cerebellum, and is possibly also associated with an imbalance in neurotransmitters (12, 14). A presumptive diagnosis of IGTS can be made based on signalment, clinical signs, neurologic examination findings, and exclusion of other potential causes of whole-body tremors. Magnetic resonance imaging (MRI) results are usually normal in IGTS patients, and cerebrospinal fluid (CSF) analysis revealed pleocytosis in a limited number of cases (7). Although considered to have an overall good outcome, relapsing and persistent mild clinical signs have been described (7, 15). Currently the syndrome is still considered idiopathic but based on the positive response to immunomodulatory drugs (namely corticosteroids), an immune-mediated etiology is suspected (7). Apart from that, little is known about the underlying etiology of IGTS and until recently, there have been no larger studies reporting on treatment and long-term outcome of this disease (7).

In human medicine, description of numerous autoantibodies involved in certain non-infectious neurologic disorders has revolutionized their understanding of their pathophysiology, their diagnosis and treatment, with some of these disorders associated with tremor syndromes (3, 7, 16–18). Tremors in people have not been described as an isolated manifestation in antibody-mediated disorders in humans, but can be part of a wider encephalopathic picture in association with leucine-rich-glioma-inactivated 1 (LGI1)/Contactin-associated protein-like 2 (CASPR2), N-methyl-D-aspartate receptor (NMDAR) and Dipeptidyl-Peptidase–Like Protein-6 (DPPX) autoantibodies or part of the presentation of certain forms of meningoencephalomyelitis with GFAP autoantibodies (18). Tremor, in conjunction with myoclonus, ataxia and autonomic dysfunction, is reported in humans with glial fibrillary acid protein (GFAP) astrocytopathy, a disease which also shows responsiveness to immunomodulatory therapy with corticosteroids and resembles necrotizing encephalitis reported in pugs (16, 19). The clinical signs of IGTS, as well as the response to immunomodulatory treatment, are more similar to other syndromes reported in humans such as acute cerebellitis, acute-cerebellar ataxia in children and opsoclonus-myoclonus syndrome (7, 20). In veterinary medicine, autoantibodies against GABA-A-R, GFAP, NMDAR and voltage-gated potassium channel (VGKC) complex have been described in various species with different neurological entities, but no autoantibodies have been so far described in dogs with IGTS (3, 17, 19, 21–24). A hypothesis that has been previously suggested is that this immune mediated disease is mediated against cells metabolizing tyrosine to produce neurotransmitters or against melanin producing cells as described in Vogt-Koyanagi-Harada syndrome (14, 25).

In the current study, it was hypothesized that autoantibodies to neuronal surface proteins would be present in serum and/or CSF samples of dogs clinically diagnosed with IGTS. The objective of the study was to describe the clinical presentation, course, and outcome of dogs with this disease and correlate potential findings with the presence or absence of autoantibodies associated with autoimmune cerebellar syndromes.

## Materials and methods

This was a retrospective multicenter study. Digital medical records from four veterinary institutions (one German teaching hospital, one United Kingdom teaching hospital, one German private clinic and one UK private clinic) were searched for dogs presumptively diagnosed with IGTS from 2015 to 2022. Inclusion criteria required the dogs to have had complete medical records for analysis and an unremarkable MRI study of the brain, as formerly described (7). Dogs with presumed toxic causes of tremor, as well as dogs with bloodwork changes deemed to be the potential cause for tremors, were excluded from study entry.

Information on signalment, history, clinical signs, complete blood count (CBC) findings, cerebrospinal fluid (CSF) analysis, serum, and CSF levels of immunoglobulin A (IgA), testing for regional infectious diseases (serology and polymerase chain reaction [PCR] of CSF for *Toxoplasma gondii*, *Neospora caninum* and Canine Distemper Virus performed at Laboklin [Bad Kissingen, Germany]), C-reactive protein (CRP) levels in CSF determined at Idexx Laboratories (Kornwestheim, Germany) or Laboklin, as well as treatment regimen, outcome and relapses were recorded.

When available, serum and CSF samples (stored until analysis at −80 degrees Celsius and transported on dry ice) were analyzed for neural antibodies at Laboratory Krone (Bad Salzuflen, Germany) and the analysis included a standard panel used to screen for autoantibodies against targets associated with autoimmune encephalitic diseases of humans consisting of:

1. Cell based assay (indirect immunofluorescence (IIF), human embryonic kidney, Euroimmun, Lübeck, Germany) for antibodies against LGl1, CASPR2, NMDAR, glycine receptor (GlyR), Anti-immunoglobulin-like cell adhesion molecule 5 (IgLON5), α-amino-3-hydroxy-5-methyl-4-isoxazolepropionic acid receptors (AMPAR1/2), Gamma-amino butyric acid receptor, type B receptors (GABABR), Gamma-amino butyric acid receptor, type A receptors (GABAAR), glutamic acid decarboxylase 65-kilodalton isoform (GAD65), DPPX, metabotropic glutamate receptor 5 (mGluR5), and Metabotropic glutamate receptor 1 (mGluR1).
2. Tissue-based assay (IIF, mouse brain tissue, Euroimmun, Lübeck, Germany) for onconeural antibodies and antibodies against neuropil, ANNA3, Purkinje cells, GAD65, GFAP, Adenylate kinase 5, Neurexin-3-alpha.

Human samples with different antibody reactivities served as positive control (26).

The onset of clinical signs was reported in days. Hyperthermia was defined as a rectal temperature greater than 39.2°C (27). Magnetic resonance imaging sequences performed varied between institutions but always included T2- weighted images (T2W) (at least in transverse and sagittal planes), fluid attenuation inversion recovery (FLAIR) and pre- and post-contrast (intravenous injection of 0.1 mmol/kg gadopentetate dimeglumine) T1- weighted images (T1W). High field MRI scanners were used: 1,5 T Magnetom Essenza (Siemens, Munich, Germany) for Glasgow University, 1.5 T Intera and 1.5 Achieva (Philips, Best, The Netherlands) for Anderson Moores, 1.5 T (Toshiba, Tokyo, Japan) for Hofheim and 3.0 T Achieva, (Philips, Best, The Netherlands) for Hannover University. Cerebrospinal fluid collection was performed under the same general anesthesia as for the MRI and analysis comprised a red blood cell count, total nucleated cell count (TNCC), protein concentration measurement and a cytological examination with a differential cell count. Normal CSF TNCC was defined as less than 5 cells/μl, and normal CSF total protein was defined as less than 25 mg/dL (cerebellomedullary cistern) (12). Cytoalbuminologic dissociation was defined as disproportionate increase in protein with a normal TNCC (12). Normal CSF IgA levels were defined as less than 0.2 mg/dL, and normal serum IgA levels were defined as less than 100 mg/dL. Follow-up time was measured in months and outcome was defined as “good” (no relapses), “acceptable” (one or more relapses that were easily managed with reintroduction of or dose escalation of a immunosuppressive drug), “poor” (characterized as spontaneous death or euthanasia due to IGTS), “unknown” (defined as patients lost to follow up in less than 1 month) and “unrelated” (defined as patients who died or were euthanized due to reasons other than IGTS) (28). Patients with relapses were categorized into those who had only one, two, or more that two relapses and the time of onset of the relapse was also documented. The presence of steroid-associated adverse-effects was documented if available.

Statistics were performed using Graph Pad Prism 10 (GraphPad Software, Boston, United States). Continuous data was analyzed using the unpaired t-test in case of normally distributed data or the Mann–Whitney U test for nonparametric data. Binary data was analyzed using the Fisher’s exact test. Data distribution was analyzed by the Shapiro–Wilk test. A value of *p* ≤ 0.05 was considered significant.

## Results

### Included dogs

Thirty-three dogs met the inclusion criteria for this study. Crossbreed dogs were most affected (*N* = 6), followed by Havanese dogs (*N* = 5), West Highland White terriers (*N* = 4), Cockapoos (*N* = 3), Dachshund (*N* = 2), Lhasa-Apso (*N* = 2), Border Collie (*N* = 1), Cocker Spaniel (*N* = 1), Poodle (*N* = 1), Pinscher (*N* = 1), Podenco (*N* = 1), Shih-tzu (*N* = 1), Siberian Husky (*N* = 1), Spitz (*N* = 1), Entlebucher mountain dog (*N* = 1), Irish terrier (*N* = 1) and Maltese (*N* = 1). Twenty-six of 33 affected dogs were female (14 intact and 12 neutered) and seven male (two intact and five neutered). The median age at presentation was 2 years (range 0.66–7 years, IQR 2.35 years). The median weight was 7.5 kg (range: 2.2–28 kg, IQR 4.7 kg).

### Clinical presentation

The median duration of clinical signs before presentation was 5 days (range 0–45 days, IQR 9 days). The most common clinical sign was head tremor, present in 94% dogs in this study (31/33), mostly as part of a generalized (whole body) tremor, present in 88% of the dogs in this study (29/33). Only two dogs were reported to have only a head tremor without a generalized tremor. Twenty-four dogs were reported to have an intention tremor (73%). Ataxia was noted in 27/33 dogs (82%); seven dogs were reported to have a combination of vestibular and cerebellar ataxia, 12 dogs had a purely cerebellar ataxia, and eight dogs had a purely vestibular ataxia. Menace response deficits were reported in 14 dogs, hypermetria in 12 dogs, a head tilt in 11 dogs, a pathologic spontaneous nystagmus in 10 dogs, and a reduced pupillary light reflex in five dogs. Epileptic seizures were reported in one dog in this study. Hyperthermia was noted in seven dogs (21%), with a mean rectal temperature of 39.5°C (range 39.3–40.3). Complete blood count changes included leukopenia (*n* = 1), hemoconcentration (*n* = 3), thrombocytosis (*n* = 1), and thrombocytopenia (*n* = 1).

### Cerebrospinal fluid analysis

Cerebrospinal fluid was sampled in all dogs in this study and was deemed normal in 20 dogs (61%). A pleocytosis was documented in 13/33 dogs; of these, seven showed a lymphocytic and six dogs had a mixed pleocytosis. The median TNCC was 3 cells/μl (range: 0–266, IQR 14.5). The median CSF red blood cell (RBC) count was 3 cells/μl (range: 0–428, IQR: 30). The median CSF protein concentration was 14.5 mg/dL (range 6.2–102 mg/dL, IQR: 6.65) and this was increased in three animals. Cytoalbuminologic dissociation was not reported in any of the dogs. CSF-IgA levels were determined in 17/33 dogs and were increased in 9 of these samples (median: 0.63, range: 0.24–2.6, IQR: 0.935). Serum IgA levels were determined in 13/33, and of these, levels were increased in six dogs (median: 176.85, range: 137.3–578.4, IQR: 125.8). Five dogs had increased levels in paired CSF and serum IgA samples. Cerebrospinal fluid C-reactive protein (CRP) levels were determined in two dogs and were increased in both samples. One of these dogs also had an increased CSF IgA level.

### Ancillary diagnostic methods

Serology for *Neospora caninum, Toxoplasma gondii* and CDV was performed in 21 dogs, and results revealed mildly increased levels of antibodies in six dogs. These were increased titers of IgG antibodies to *Toxoplasma gondii (1:200 in one dog, and 1:128 in another dog, reference range 1:100) and increased titer of antibodies to CDV (1,40 in one dog, 1:80 in four dogs, reference range < 1:20).* One dog had simultaneously increased titers to both *Toxoplasma gondii* and CDV. PCR assays on CSF for *Neospora caninum, Toxoplasma gondii* and CDV were performed in 13 dogs, and all were negative. All dogs with increased serum antibody titers to *Toxoplasma gondii* or CDV had negative PCR results to these pathogens in the paired CSF samples. PCR for Antigen Receptor Rearrangements (PARR) was performed on the CSF sample of one dog and was unremarkable.

### Neural autoantibodies

Screening for neural antibodies was performed on serum and/or CSF samples in 30 of the 33 dogs. Analysis of CSF and serum was not possible due to insufficient sample quantity in three dogs. The analysis showed an increased titer of mGluR1 antibodies in two dogs, GFAP and mGLuR1 antibodies in one dog and an increase in unspecific autoantibodies which could not be further classified in two dogs. Of the three dogs with an increase in mGluR1 antibodies, one dog had an increased serum (1:160) and CSF (1:2) titer of these IgG neural antibodies in the cell-based assays. There was no corresponding staining pattern noted on the tissue-based assay. A repeated blood sampling and CSF-tap 3 months after initiation of immune-suppressive therapy revealed only a lower, but still increased serum titer (1:40) with normalization of the CSF titer. One dog had an increase in the CSF GFAP antibodies titer (>1:2) correlating to the tissue-based assay as fluorescence of the subpial astrocytes. A repeated blood sampling and CSF tap, during a re-check after initiating treatment, showed an increase in serum mGluR1 titer (1:40) with no corresponding increase of the CSF titer and no corresponding staining pattern noted on the tissue-based assay. The third dog had an increase of serum mGluR1 antibody titer (1:40) with no corresponding increase of the CSF titer and no corresponding binding pattern noted on the tissue-based assay. Two dogs had an increase of unspecific antibodies in the tissue-based assay, which could not be further classified. There was no increase of antibodies in any serum or CSF samples noted in the cell-based assay of these two dogs.

### Treatment

All dogs received immuno-modulatory therapy; 33 dogs were treated with prednisolone and four dogs received additional immunosuppressive medications (ciclosporin *n* = 4, cytosine arabinoside *n* = 2 and mycophenolate mofetil *n* = 1). Ciclosporin was administered orally at following doses: 3.5 mg/kg two times daily in one dog, 5 mg/kg two times daily in two dogs and 7 mg/kg two times daily in one dog. Cytarabine was given as four 50 mg/m^2^ subcutaneous injections every 12 h for 48 h; a cycle repeated every 3+ weeks. Mycophenolate mofetil was given orally at 10 mg/kg two times daily. A starting dose of prednisolone of 4 mg/kg/day was given to two dogs, 2 mg/kg/day to 23 dogs and 1 mg/kg/day to eight dogs. Data on the course of prednisolone tapering was available for 23 dogs (70%). Prednisolone was tapered in less than 6 months in 19 dogs, and in more than 6 months in four dogs. Prednisolone-related adverse effects were noted in 48% of dogs: lethargy (*n* = 11), polyuria/polydipsia (*n* = 10), polyphagia (*n* = 9), muscle wasting (*n* = 8), alopecia (*n* = 4), abdominal distension (*n* = 3), panting (*n* = 1) and urinary incontinence (*n* = 1). Additional drugs administered at the time of diagnosis included diazepam (*n* = 17), antibiotics (*n* = 16) and phenobarbital (*n* = 3).

### Follow-up and outcome

Median follow up time was 5 months (range: 0–100 months, IQR: 8 months). A good outcome was reported in 18/33 dogs (55%), an acceptable outcome in 8/33 dogs (24%) and the outcome was unknown in 7/33 dogs (21%, lost to follow up). None of the dogs in this study suffered a poor outcome. Of the dogs with a known outcome (26/33) 11.5% (*N* = 3) suffered at least one relapse, 7,7% (*N* = 2) relapsed twice, and 11.5% (*N* = 3) relapsed more than two times. Supplementary Table S1 shows information on signalment, clinical presentation, diagnostic findings, therapy, outcome and corticosteroid adverse-effects of all dogs in this study. Supplementary materials S1 shows detailed information on signalment, clinical presentation, diagnostic findings, therapy, outcome and corticosteroid adverse-effects of all dogs with detected neural autoantibodies in this study.

### Statistical analysis

No statistically significant relationships were found between the outcome and the following parameters: age at presentation, sex, weight, duration of clinical signs before presentation, abnormalities on neurologic examination, pleocytosis, serum or CSF-IgA concentration, starting prednisolone dose, duration of prednisolone tapering, use of diazepam or antibiotics, presence of prednisolone side-effects. There were significantly fewer dogs with distemper antibodies (*N* = 1, *p* = 0.0476) and dogs that received ciclosporin (*N* = 1, *p* = 0.0468) in the good-outcome group than in the acceptable-outcome group (*N* = 4, and *N* = 3 respectively). Animals in the good-outcome group received significantly higher doses of diazepam than animals in the acceptable-group (mean 0.63 mg/kg TID, range: 0.2–1.5 mg/kg TID, IQR:0.42, *p* = 0.0075). Also, there was less muscle wasting (*N* = 1, *p* = 0.0062) and alopecia (*N* = 1, *p* = 0.0041) observed (as prednisolone adverse-effects) in the group with good outcome, in comparison to the acceptable outcome group (*N* = 5, and *N* = 3 respectively). The different features are depicted in Table 1. Furthermore, a positive correlation was seen between the age at presentation and the duration of clinical signs before the presentation (*r* = 0.498, *p* = 0.003) and to the CSF protein concentration (*r* = 0.421, *p* = 0.016). A positive correlation was also noted between the prednisolone dose and the CSF TNCC (*r* = 0.394, *p* = 0.023), and between serum IgA and CSF IgA concentrations (*r* = 0.939, *p* < 0.0001). A negative correlation was noted between the prednisolone dose and the CSF red blood cell count (*r* = −0.428, *p* = 0015).

**Table 1:** Summary of statistically significant differences between dogs without and those with relapses of idiopathic generalized tremor syndrome.

	Good outcome	Relapses	*p*-value
Positive distemper antibody titer	*N* = 1	*N* = 4	0.0476
Ciclosporin administration	*N* = 1	*N* = 3	0.0468
Diazepam dose	Mean 0.63 mg/kg TID	Mean 0.59 mg/kg TID	0.0075
Prednisolone adverse effects (muscle wasting and alopecia)	*N* = 1 (muscle wasting) *N* = 1 (alopecia)	*N* = 5 (muscle wasting) *N* = 3 (alopecia)	0.0062 (muscle wasting) 0.0041 (alopecia)

A larger proportion of dogs with neural autoantibodies presented with hyperthermia compared to the group of dogs without detectable neural autoantibodies (*p* = 0.0679). All dogs positive for neural autoantibodies had an abnormal CSF finding (either pleocytosis, or pleocytosis and increased protein concentration), and dogs with positive neural antibody titers had significantly more CSF nucleated cells per mcL (mean: 71.6, range: 18–266, IQR: 129.5) than dogs with no neural autoantibodies (mean: 8.7, range: 0–105, IQR: 5; *p* = 0.0032). There was also a larger proportion of dogs with a lymphocytic pleocytosis in the neural autoantibody-positive group compared to the negative group (*p* = 0.0679). A significantly larger proportion of dogs received ciclosporin in the group with positive neural antibodies compared to the antibody negative group, in which no dog received ciclosporin (*p* = 0.023). The distinct findings between dogs tested positive or negative for neural autoantibodies are depicted in Table 2.

**Table 2:** Summary of distinct findings between dogs with and those without detectable neural autoantibodies in idiopathic generalized tremor syndrome.

	Neural autoantibodies positive (5/30 dogs)	Neural autoantibody negative (25/30 dogs)	*p*-value
Hyperthermia	60% (*N* = 3)	16% (*N* = 4)	0.0679
CSF pleocytosis +/− increased protein	100% (*N* = 5)	24% (*N* = 6)	
Mean CSF NCC/mcL	71.6	8.7	0.0032
Lymphocytic pleocytosis	60% (*N* = 3)	16% (*N* = 4)	0.0679
Ciclosporin administration	40% (*N* = 2)	0% (*N* = 0)	0.023

## Discussion

This is the first study examining the presence of serum and CSF neural autoantibodies in 30 dogs clinically diagnosed with IGTS. The results indicate a sporadic increase in certain neural antibodies, namely mGluR1 in two dogs, GFAP and mGLuR1 in one dog and unspecific antibodies in two dogs, possibly related with a more severe form of IGTS, meriting further investigation for their role in this disease process.

Diseases associated with neural autoantibodies are increasingly recognized in human and, more recently, veterinary medicine (3, 16, 17, 19, 21, 23, 24). Since their first description almost 20 years ago, numerous antibodies that target either the neuronal cell surface proteins or intracellular neuronal proteins have been described (29). Currently, there are almost 30 different neural antibodies described in human medicine, and their role in a plethora of different neurological syndromes has been proven (30). These range from clinical entities presenting as epileptic seizure disorders, cognitive decline, psychiatric disorders, all the way to ataxia and even autonomic or myasthenic disorders (29). Antineuronal antibodies in human medicine are classified into antibodies against intracellular antigens (frequently of paraneoplastic origin) and those against neuronal surface antigens with recommended laboratory diagnostics following a two steps strategy consisting of a first tier screening technique (indirect immunofluorescence of immunohistochemistry) performed on animal neural tissue (tissue-based assays) to detect possible reactivity and identify a characteristic pattern, if present, followed by a confirmatory analysis with different methodologies accordingly to the type of antibody investigated (antibodies against intracellular vs. cell surface antigens) (30). Neural autoantibodies have also been noted in veterinary medicine with increasing frequency over the last years. One of the first, and most famous cases, gaining a lot of media coverage, was the case of “Knut,” a polar bear, who was post-mortem diagnosed with anti-NMDR encephalitis (21). After “Knut’s” diagnosis, neural antibodies have been described in cats with limbic encephalitis (LGl1), dogs with meningoencephalitis of unknown origin (MUO) (NMDAR), necrotizing encephalitis (anti-astrocytic GFAP) and one dog presenting with acute epileptic seizures and erratic behavioral changes (GABAA-R) (3, 17, 21, 22, 24).

The data regarding signalment, clinical presentation, laboratory parameters and ancillary diagnostics of dogs with IGTS gathered in this study confirm what has already been reported in previous studies (7, 10, 11, 15). Statistical analysis revealed a tendency of dogs tested positive for neural autoantibodies to present with hyperthermia, as an additional systemic sign to the commonly reported vestibulo-cerebellar dysfunction. Seven dogs (21%) in this study had an increased rectal temperature, a finding also reported in a recent study, where hyperthermia was noted in 22.6% of the patients presenting with IGTS (7). Although fever is usually associated with infectious encephalitis in people, it is important to note, that up to 50% of people suffering from autoimmune encephalitis will present with or develop fever during the course of the disease (31). It is possible that a subgroup of dogs with IGTS suffer from a more severe form, characterized by development of systemic signs. Seventy percent of humans with anti-NMDA receptor encephalitis initially depict symptoms similar to a cold, such as nausea, fever, headache, and fatigue (32).

Of note, 79% of the dogs in our study were female and a female bias has already been reported for this disease in other studies (7, 15). This finding has also been described in other immune-mediated diseases affecting dogs, such as MUO and immune-mediated hemolytic anemia (1, 33). Acute cerebellitis, a rare entity in human medicine, is also noted to have a female predisposition, with van Samkar et al. reporting that 63% of the adult patients presenting with this disease were women (34). Autoimmune disorders are characterized as a condition in which the host’s immune system mistakenly attacks itself, and, in human medicine, a gender bias with an increased prevalence among women, occurring at a rate of 2 to 1, has been described (35). Although the reason for the female sex predilection in animal and human autoimmune disease is unknown, the influence of the difference between sex chromosomes in males and females as well as the difference between sex hormones and pregnancy have all been suggested to explain this discrepancy (35). Although a prevalent feature of autoimmune disease in humans, a female bias has so far not been noted in other canine immune-mediated diseases such as steroid responsive meningitis arteritis (SRMA), immune-mediated polyarthritis or allergic disease (36–38). It is possible that both breed and sex play a role in the etiopathogenesis of IGTS.

Tremors are the hallmark of IGTS, but tremor without other concurrent neurological abnormalities is uncommonly seen in immune mediated diseases in human medicine (7). Tremor in conjunction with myoclonus and ataxia is reported in the syndrome of glial fibrillary acid protein (GFAP) astrocytopathy in humans (16). GFAP antibodies were found only in one dog in this study. Interestingly, GFAP antibodies have also been reported in pugs with necrotizing encephalitis, a disease, which, like the GFAP astrocytopathy in humans, MUO and IGTS in dogs, are all, reported to be, corticosteroid responsive to some degree (16, 17).

Cerebrospinal fluid abnormalities were detected in almost 40% of the dogs in our study, including all dogs with detectable neural antibodies. Pleocytosis (mixed or lymphocytic) was the most prevalent CSF abnormality. This is in accordance with the study performed by Phillipps et al.(2022), in which a pleocytosis was noted in half of the patients (7). A tendency was noted, for dogs with positive neural antibodies to have a lymphocytic pleocytosis. A mild mononuclear or lymphocytic pleocytosis has also been commonly reported in other studies on IGTS (6, 9, 11, 15). Pleocytosis was also noted in 44% of human patients with anti-mGluR1 encephalitis in a larger study dealing with clinical features of this disease (39). A positive correlation was noted between the prednisolone dose used and the CSF TNCC, indicating that clinicians tended to use higher initial doses of immune-suppressive drugs when treating dogs with a more pronounced pleocytosis. This probably reflects an underlying consternation that such cases have a more severe form of the disease. The broad spectrum of diverse signalments, clinical signs and differences in the results of CSF analysis could point to the fact that there is more than one disease under the umbrella-term IGTS, or that the autoantibodies occur only at a certain stage.

Cerebrospinal fluid IgA levels were measured in around half of the dogs in this study (17/33) and were increased in just over half of these samples (9/17). Serum IgA levels were determined in 13/33 dogs, and of these, six were increased. To the author’s knowledge, this is the first study which reports increased CSF and serum IgA in dogs with IGTS. CNS inflammation triggers non-specific production of different Ig classes and, while the role of IgA in the CNS is not completely understood, its production does not seem to be specific for any particular antigenic stimulus (40). IgA concentrations have been found to be increased in CSF and serum of dogs with SRMA, and in single cases with different inflammatory CNS diseases, while in certain neoplastic diseases or most inflammatory diseases IgA concentrations were only increased in the CSF (40, 41). Five dogs in our study had a simultaneous increase in CSF and serum IgA concentrations. This could indicate, that IGTS like SRMA also has a systemic inflammatory component, a theory already proposed by Phillipps et al. (7).

The current study represents the first report of circulating and CSF mGluR1 antibodies identified in individual dogs with an immune mediated neurological disease. However, its potential relationship to the disease IGTS still needs to be further investigated. Metabotropic glutamate receptor 1 is a G-protein-coupled receptor, activation of which facilitates long-term depression of parallel fiber–Purkinje cell synapses critical for cerebellar motor learning (42). In human medicine, mGluR1–immunoglobulin G (IgG) autoantibody is a biomarker of autoimmune cerebellar ataxia, reported in a paraneoplastic context (usually lymphoma) or without a neoplasm being detected (42). Anti-mGluR1 encephalitis in human medicine manifests as a severe, subacute cerebellar syndrome with behavioral/cognitive changes, often resulting in long-term disability and cerebellar atrophy (39, 42). This contrasts with IGTS, where tremors are the most obvious clinical sign, and the disease has a good prognosis, although relapses are not uncommon (30.8% of dogs for which outcome was available in this study).

All the dogs in this study, which had neural antibodies detected had a CSF pleocytosis, and those that were positive for neural autoantibodies had significantly more CSF cells per microliter than dogs without detectable antibodies. A tendency (although not statistically significant) was also seen between dogs positive for neural autoantibodies and the CSF protein concentration. Pleocytosis was also noted in the two dogs with identified unspecific neural antibodies. The dog with the repeated blood and CSF sampling (3 months after the initiation of the immunosuppressive therapy) that showed a decrease of the serum and CSF neural antibodies had normal CSF results after initiating treatment. The other dog with increased GFAP antibodies in the first CSF sample and a pleocytosis at that time, had a low titer of serum mGluR1 antibodies and no pleocytosis in the second CSF sample. Cerebrospinal fluid and MRI findings are frequently normal in people with anti-mGluR1 encephalitis, however, some patients have an acute presentation, usually accompanied by significantly higher CSF pleocytosis compared to those with the more frequent, subacute onset (39). It is therefore possible that there are several forms of IGTS in dogs, and the ones accompanied by a more severe CSF pleocytosis also could be associated with measurable autoantibodies, which could be involved in the pathogenesis of this disease.

A good outcome was noted in more than half of the dogs in this study, while a poor outcome was not noted in any of the dogs in our study. However, it is important to mention, that seven dogs were lost to follow-up and eight dogs suffered one or more relapses, as described in a recent study (7). Relapses were only noted in dogs from the Hannover center, where only five dogs had no relapses and eight dogs had relapses. It is possible that this disease has a geographical difference, or that the owners in the United Kingdom are more reluctant to seek out specialized veterinarians in case of relapses, especially since relapses were previously considered uncommon in dogs with IGTS (6) Our study found no statistically significant difference between dogs with or without relapses and clinical presentation, starting dose of prednisolone and the pace at which prednisolone was tapered. Also, in dogs with relapses, a favorable response was always noted upon reinstitution of corticosteroid therapy.

Limitations of this study are:

1. Multicenter retrospective study: it makes it harder to gather precise information about the different treatment regimens and diagnostic procedures used.
2. No histopathological confirmation: since there is no definitive ante-mortem test for IGTS, and although care was taken to eliminate toxic, metabolic, infectious, and other structural causes, these cannot be completely ruled out due to absence of histopathological confirmation.
3. The use of human immune assays for detection of antibodies to new antigenic targets in dogs is significantly limited by the lack of canine positive controls, as previously described in a study investigating the presence of specific neural antibodies in dogs with epilepsy or dyskinesia (23). Multiple studies point to the need for the development of (canine) species-specific assays for neural antibody detection and also the need for inclusion of control groups to assess whether there is a significant association with disease (23, 43). Therefore, it could be hypothesized that autoantibodies, which are at this time rarely detected in dogs with IGTS using the current diagnostic technique, could be detected even more frequently if species specific methods are used. A larger prospective study using species-specific autoantibodies, which are possibly still not detected in dogs with this disease, is warranted to help improve our understanding of why some dogs suffer relapses and improve the diagnostics plan and treatment strategies of this condition.
4. It is possible that the test itself is not ideal for detecting neural autoantibodies, as demonstrated in humans: human patients with autoimmune encephalitis do not have any identifiable autoantibodies (seronegative) representing a disease category with novel, yet to be identified antibodies or T-cell mediated disease (29, 44). Most of the dogs in our study did not have any identifiable autoantibodies, and two dogs had autoantibodies which could not be classified. It is possible that there are undetected autoantibodies in dogs with this disease, or that this disease is primarily mediated through T-cells as described in Vogt-Koyanagi-Harada syndrome and therefore are not associated with increased neural autoantibodies.
5. The detected neural autoantibodies may not represent a relative finding for IGTS: as already demonstrated in human medicine, the finding of neural autoantibodies should be considered in the context of the presenting clinical signs as their presence does not always equate and immune-mediated neurological disease and their presence can indicate the presence of other disease (such as cancer without neurological disease) (30).

In conclusion, IGTS is proposed to be an immune-mediated disorder of the CNS, which is potentially mediated by neural antibodies in a subgroup of patients. There are still large gaps in the current understanding of the etiopathogenesis of this disease, and research on this topic is relatively sparse, probably due to the good prognosis and straight-forward treatment possibilities.

## Data Availability

The original contributions presented in the study are included in the article/Supplementary material, further inquiries can be directed to the corresponding author/s.
