# Supplementary material for: Canine idiopathic generalized tremor syndrome, immune-mediated?
Source: Front Vet Sci. 2024 Sep 20;11:1453698. doi: 10.3389/fvets.2024.1453698 (PMC11449864; doi:10.3389/fvets.2024.1453698)
Supplement: Supplementary file 2 [file Table_1.DOCX]

**Supplementary Table 1.** Detailed information on signalment, clinical presentation, diagnostic findings, therapy, outcome and corticosteroid adverse-effects of all dogs in this study.

| **No** | **Age at  pres.**  **(years)** | **Breed** | **Sex** | **Weight**  **(kg)** | **Neural Ab** | **Duration  of signs  before**  **presentation**  **(days)** | **Hyperthermia (temperature in Celsius)** | **CSF  abnormalities** | **Distemper antibodies**  **(normal**  **< 1:20)** | **Ciclosporin**  **administered** | **Diazepam  dose**  **(mg/kg TID)** | **Outcome** | **Steroid**  **adverse**  **effects** |
| --- | --- | --- | --- | --- | --- | --- | --- | --- | --- | --- | --- | --- | --- |
| **1** | 7 | Spitz | F | 10 | no material | 45 | no | yes | Not done | no | NA | unknown | no |
| **2** | 2 | Entelbucher mountain dog | F | 22 | negative | 7 | yes (39.3) | no | 0 | no | NA | good | no |
| **3** | 2 | Lhasa Apso | F | 4.6 | negative | 21 | no | yes | Not done | no | NA | relapse (once) | NA |
| **4** | 1 | Havanese | F | 5 | negative | 3 | no | no | Not done | no | 2 | relapse (once) | NA |
| **5** | 1 | Border Collie | FN | 28 | negative | 5 | no | no | Not done | no | 0,2 | relapse (twice) | NA |
| **6** | 3.5 | Havanese | MN | 6.7 | positive | 7 | no | yes | Not done | yes | NA | relapse (> twice) | yes |
| **7** | 3.5 | Pinscher | F | 2.2 | negative | 14 | yes (39.6) | yes | 0 | no | NA | good | no |
| **8** | 1 | West Highland White terrier | F | 5.5 | negative | 8 | no | no | 0 | no | 1 | good | NA |
| **9** | 3.5 | Mixed | M | 3.9 | negative | 1 | no | no | Not done | no | 0.5 | unknown | NA |
| **10** | 4 | Podenco | FN | 8.8 | negative | 14 | no | no | Not done | no | NA | relapse (> twice) | yes |
| **11** | 2 | Mixed | FN | 8.2 | no material | 1 | no | yes | 1:80 | yes | 0,5 | relapse (> twice) | yes |
| **12** | 4 | Mixed | FN | 8.2 | negative | 22 | yes (39.3) | no | Not done | no | 0.5 | relapse (once) | yes |
| **13** | 1 | West Highland White terrier | F | 5.8 | negative | 10 | no | no | 1:80 | no | NA | unknown | NA |
| **14** | 4.5 | Irish terrier | F | 10.5 | positive | 1 | yes (39.3) | yes | 1:80 | yes | NA | good | yes |
| **15** | 2 | Mixed | F | 2.9 | negative | 3 | no | yes | Not done | no | NA | unknown | NA |
| **16** | 2 | Havanese | FN | 5.6 | negative | 3 | yes (39.4) | no | Not done | no | NA | good | yes |
| **17** | 1 | West Highland White terrier | F | 7.3 | no material | 0 | no | no | 1:40 | yes | 0.25 | relapse (twice) | yes |
| **18** | 1.5 | Havanese | F | 3.6 | negative | 7 | no | no | 1:80 | no | 0.1 | relapse (once) | yes |
| **19** | 1.6 | Miniature poodle | M | 3.4 | negative | 21 | no | no | 0 | no | NA | good | yes |
| **20** | 1.2 | Havanese | F | 3.5 | positive | 4 | yes (39.8) | yes | 0 | no | 1.5 | good | no |
| **21** | 0.66 | Shih-tzu | F | 7 | negative | 2 | no | no | Not done | no | NA | good | no |
| **22** | 1.6 | Cockapoo | FN | 9.4 | negative | 14 | no | no | Not done | no | 0.5 | good | yes |
| **23** | 0.8 | Mixed | FN | 10.3 | negative | 14 | no | no | Not done | no | NA | good | no |
| **24** | 1.7 | Miniature Dachshund | MN | 6 | negative | 10 | no | no | Not done | no | NA | good | yes |
| **25** | 3.2 | Cocker Spaniel | MN | 11.6 | negative | 4 | no | no | Not done | no | 0.36 | good | no |
| **26** | 2 | West Highland White terrier | FN | 7.5 | negative | 5 | no | yes | Not done | no | 0.26 | unknown | no |
| **27** | 1 | Cockapoo | FN | 9.5 | negative | 3 | no | no | Not done | no | 0.2 | unknown | no |
| **28** | 2 | Siberian Husky | F | 23 | negative | 2 | no | yes | Not done | no | 0.4 | good | yes |
| **29** | 4 | Maltese | MN | 7 | negative | 5 | no | yes | Not done | no | 0 | good | no |
| **30** | 2.2 | Mixed | FN | 7.9 | negative | 5 | no | no | Not done | no | 0.6 | good | yes |
| **31** | 1 | Cockapoo | F | 10.4 | positive | 7 | no | yes | Not done | no | 0.5 | good | yes |
| **32** | 2.3 | Dachshund | FN | 9.9 | positive | 1 | yes (40.3) | yes | Not done | no | 0 | good | yes |
| **33** | 1.6 | Lhasa-Apso | MN | 9.3 | negative | 7 | no | no | Not done | no | 0.2 | good | yes |
